# Supplementary material for: Polyketide synthase gene clusters in the ericoid mycorrhizal fungus Oidiodendron maius: genome-wide prediction, expression profiling, and functional characterization of knockout mutants
Source: Front Plant Sci. 2026 Sep 16;17:1924430. doi: 10.3389/fpls.2026.1924430 (PMC13623903; doi:10.3389/fpls.2026.1924430)
Supplement: Supplementary file 1 [file SupplementaryFile1.zip › Supplementary Materials/Supplementary Folder 1.zip!/Oidma1_AssemblyScaffolds_Repeatmasked/knownclusterblast/cluster16/jgi.p_Oidma1_18549_mibig_hits.html]

| MIBiG Protein | Description | MIBiG Cluster | MiBiG Product | % ID | % Coverage | BLAST Score | E-value |
| --- | --- | --- | --- | --- | --- | --- | --- |
| EED52435.1 | isoflavone\_reductase\_family\_protein | BGC0001306 | Asperipin\_2a\_biosynthetic\_gene\_cluster | 36 | 97.6821192053 | 168 | 1.6e-41 |
